# Are we over treating Pineal Parenchymal tumour with intermediate differentiation? Assessing the role of localised radiation therapy and literature review

**DOI:** 10.1186/s40064-015-1502-9

**Published:** 2016-01-12

**Authors:** P. Das, S. Mckinstry, A. Devadass, B. Herron, D. S. Conkey

**Affiliations:** Northern Ireland Cancer Centre, Belfast, UK; Royal Victoria Hospital, Belfast, UK

**Keywords:** Pineal Parenchymal tumour, Pineal Parenchymal tumour with intermediate differentiation (PPTID), Radiotherapy, Chemotherapy

## Abstract

Pineal Parenchymal tumour with intermediate differentiation (PPTID) is a rare disorder, first classified by World Health Organisation in 2000. There are very few published data available and optimal management is yet to be determined. Management has varied from surgery alone to craniospinal radiotherapy with or without chemotherapy. We present our experience of PPTID treated with radiotherapy alone. We conducted a retrospective review of patients who were diagnosed with PPTID and treated with radiation therapy at our institute from 2010 onwards. Between January 2010 to January 2013, 5 patients of PPTID were treated at our institute. Median age is 44 (range 24–62). All patients had preoperative MRI scan of brain and spine. Imaging did not identify any spinal dissemination. None of the patients underwent a gross total resection, due to the tumour location and technical difficulties. All patients were treated with external beam radiation therapy to primary lesion only with a dose of 54 Gy in 30 fractions after surgery. 4 patients had good partial response and the remaining 1 has stable disease. After 21.4 months of median follow up no disease recurrence was reported. So far there is no evidence of cerebral white matter abnormalities on MRI scan or neurocognitive disorders. Our experience indicated that localised radiation therapy could be an effective treatment strategy for PPTID, considering the long natural course of the disease and the late adverse effects of intensive treatment.

## Background

Tumours arising in the pineal area are heterogeneous in nature (British Neuro-Oncology Society/NCAT Rare Tumour Guidelines 2011). Pineal parenchymal tumours are rare, accounting for <0.3 % of all primary central nervous system tumours. Among all Pineal tumours PPTID is a relatively rare group with considerable morphological variation. PPTID is a new addition to the WHO classification of central nervous system tumours (Louis et al. 2007) and may account for up to 20 % of pineal tumours. The WHO classification of central nervous system tumours (2007 revision) has subdivided pineal tumours into 4 grades: Pineocytoma (PC) grade 1 and Pineoblastoma (PB) grade IV (Jouvet et al. 2000); Papillary tumor of the pineal region and pineal parenchymal tumor of intermediate differentiation (PPTID) are considered as intermediate grade and both can be subdivided into grade 2 or 3 (2).

There is no particular sex predominance and they are more common in middle aged patients (Sato and Kubota 2009). The optimal management for PPTIDs has yet to be determined. It is a relatively new disease entity and very few published data are available. At one end of the spectrum, patients treated with surgery alone, exhibited long term survival. At the other extreme, studies describe PPTIDs as tumours with seeding potential and recommend postoperative treatment in a manner similar to that for pineoblastomas (Schild et al. 1993). This raises an important question as to what should be the ideal treatment for PPTID. As it is an intermediate grade lying between pinocytoma and pinoblastoma do we need to adopt an intermediate treatment strategy? The aim of this study was to retrospectively analyse the role of localised radiation therapy for PPTID patients treated at our institution.

## Methods

We conducted a retrospective review of patients who were diagnosed with PPTID and treated with radiation therapy at our institution from January 2010 onwards. The patients were identified from an in-house data base. Clinical data, pathological results, imaging [magnetic resonance imaging (MRI)], initial treatment, resection status, details of radiation therapy, initial response to treatment, recurrence pattern and late adverse toxicities, were collected. The study was ethically approved by Department of Oncology and Neurosurgery. All patients were contacted and consent was obtained.

‘Gross total resection’ was defined as no evidence of contrast-enhancing tumour on postoperative images; ‘R2-resection’ was any surgical tumour resection less than gross total resection; and ‘biopsy only’ was no surgical tumour resection due to inoperability, with a biopsy performed to determine tumour histology (Stoiber et al. 2010).

The response criteria were described as follows: complete response (CR), disappearance of tumour; partial response (PR), >50 % decrease in tumour size; progressive disease, >25 % increase in tumour size or any appearance of new sites; stable disease (SD), all other situations.

The late effect of the treatment were assessed by appearance of cerebral white matter abnormalities in MRI after treatment and grading system of Radiation Therapy Oncology Group (RTOG) and common terminology criteria for adverse events (CTCAE), version 4.0. The classification according to neurocognitive disorders are as follows: RTOG (neurological/cortical) grade 1, mild somnolence or agitation; grade 2, moderate somnolence or agitation; grade 3, severe somnolence, agitation, confusion, disorientation or hallucinations; and grade 4, coma, seizures and toxic paralysis; and CTCAE (cognitive disturbance) grade 1, mild cognitive disability, not interfering with work/school/life performance, specialized educational services/devices not indicated; grade 2, moderate cognitive disability, interfering with work/school/life performance but capable of independent living, specialized resources indicated on a part-time basis; grade 3, severe cognitive disability, with significant impairment of work/school/life performance.

### Patient demographics

**Fig. 1:**
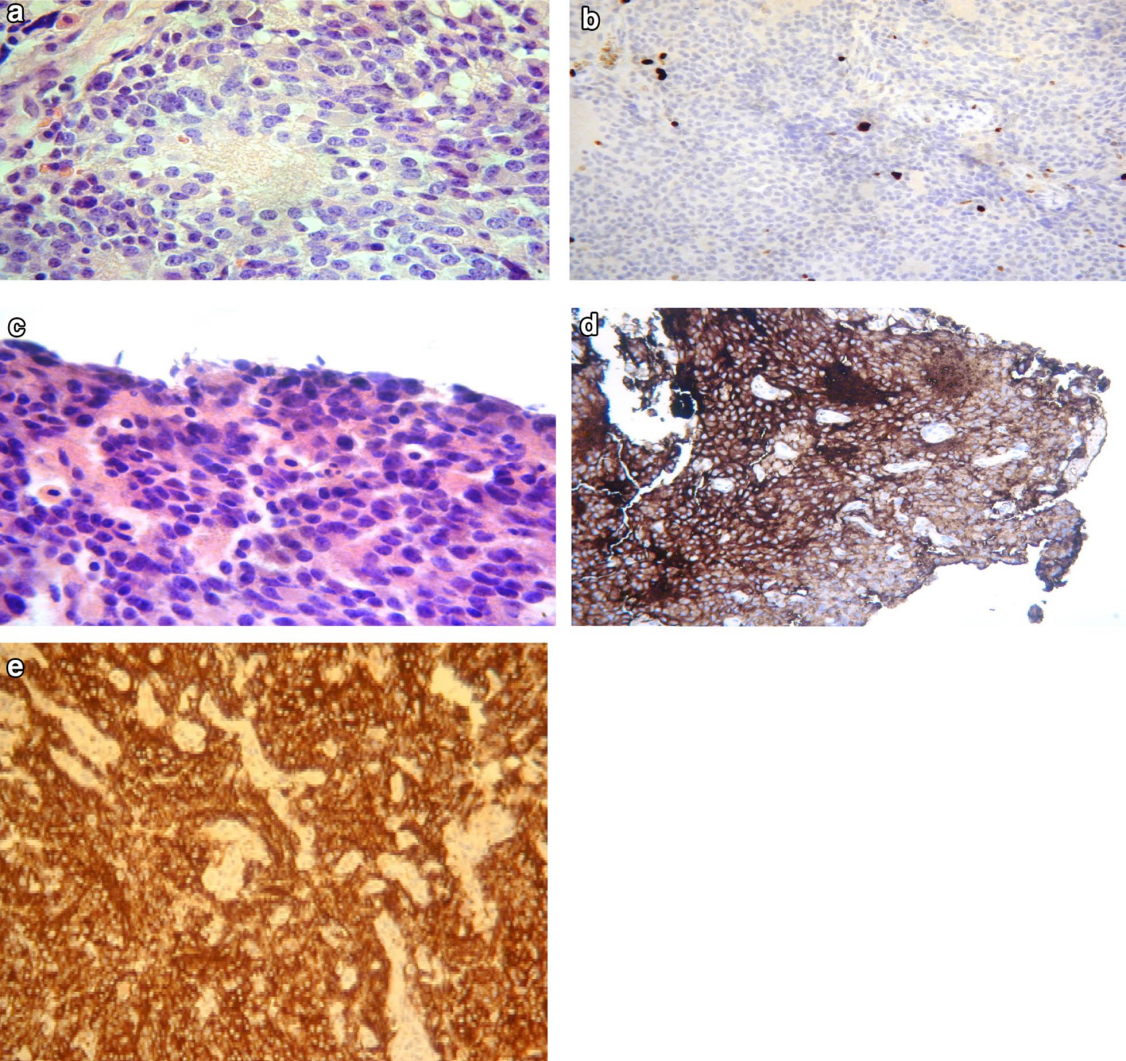
**a** Malignant pineocytes with stippled nuclear chromatin and moderately eosinophilic cytoplasm arranged around a Homer-Wright rosette.                         **b** MIB-1 demonstrating a low proliferative index. **c** Frozen section image: tumour appear more closely packed with denser chromatin. Apoptotic bodies are visible (*arrow*). **d** Synaptophysin immunochemistry showing granular cytoplasomic positivity. **e** Strong neurofilament staining on all cells

**Fig. 2:**
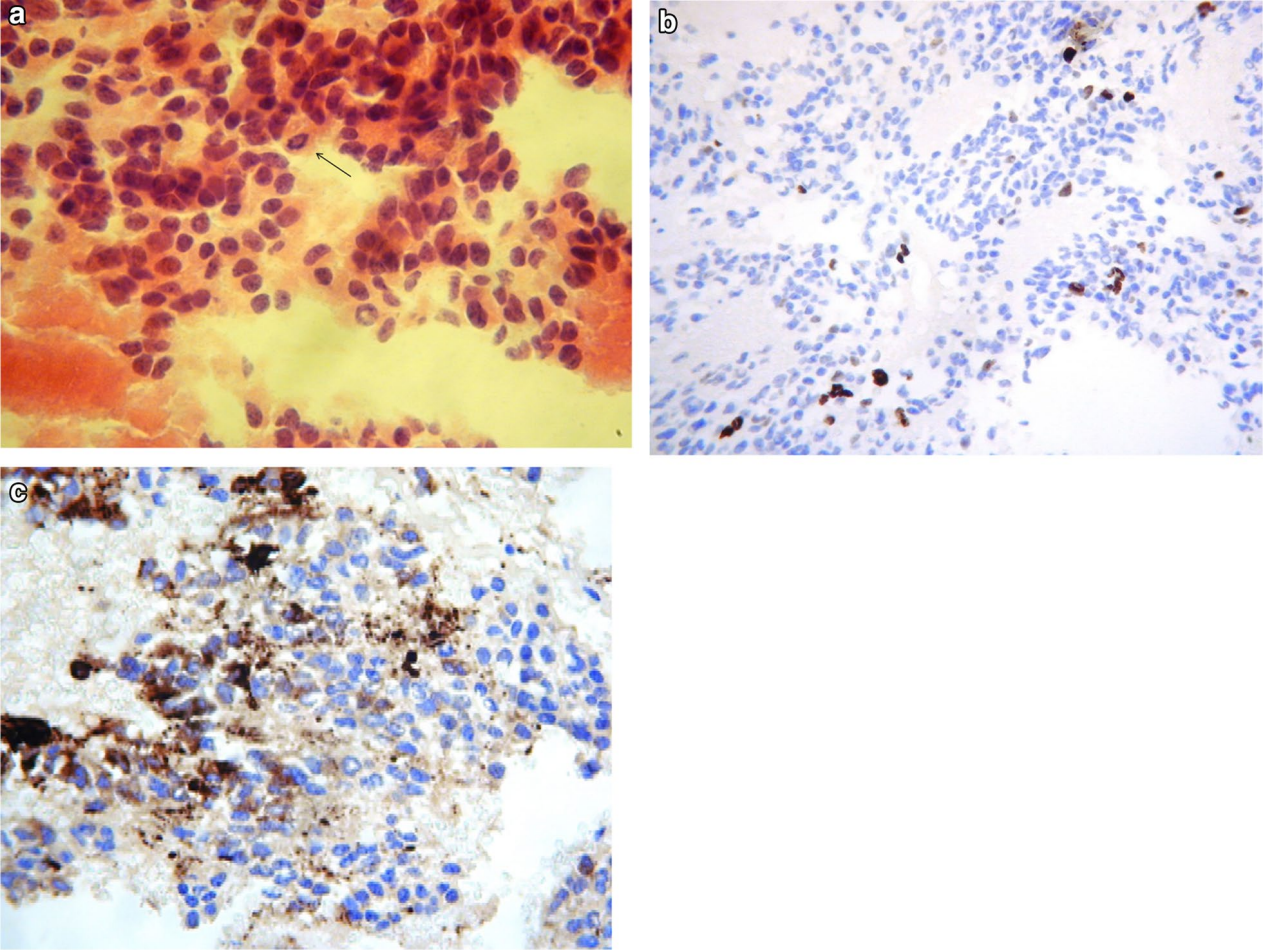
**a** Malignant pineocytes exhibit increased nuclear pleomorphism. A mitotic figure is present (*arrow*). **b** MIB-1 demonstrating higher proliferative index. **c** Weak, patchy neurofilament staining positivity most cells are negative

Between January 2010 and January 2013, 5 patients with PPTID were treated at our institution. Table 1 summarizes the patient and tumour characteristics, and the treatment received. Median age was 44 (range 24–62) and 60 % are female. All patients had preoperative MRI scan of brain and spine. Imaging did not identify any spinal dissemination. No patient had routine lumbar puncture preoperatively. Two patients were pathologically classified as WHO grade III and 3 were grade II. The grade of the disease was determined by the mitotic count, presence of neurofilament staining and the proliferation index (Figs. 1, 2).

**Table 1:** Patients and tumour characteristics

Patients	Sex	Age	CSF dissemination	Completeness of surgery	Grade	Neurofilament staining	Proliferative index (%)	Response	Recurrence
1	F	37	No	Biopsy only	3	+	10–15	PR	No
2	F	61	No	R2	2	++	0	SD	No
3	F	56	No	R2	2	+++	3	PR	No
4	M	24	No	R2	3	±	10–12	PR	No
5	M	62	No	R2	2	+++	5	PR	No

**Fig. 3:**
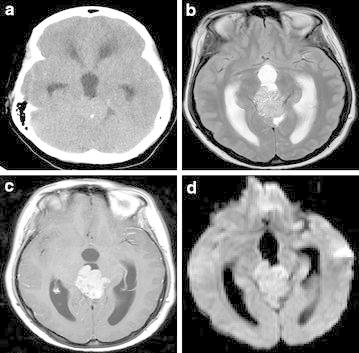
CT scan **a** shows homogenous mass in pineal region with peripheral calcification and obstructive hydrocephalus. **b** T2-weighted MRI shows multiple small cysts within the mass and probable invasion of left thalamus, **c** confirmed on T1-weighted scan after intravenous Gadolinium. **d** Diffusion-weighted scan (*b* = 1000) shows small area of diffusion restriction anteriorly within the tumour (confirmed on Apparent Diffusion Coefficient map), close to the left thalamus

**Fig. 4:**
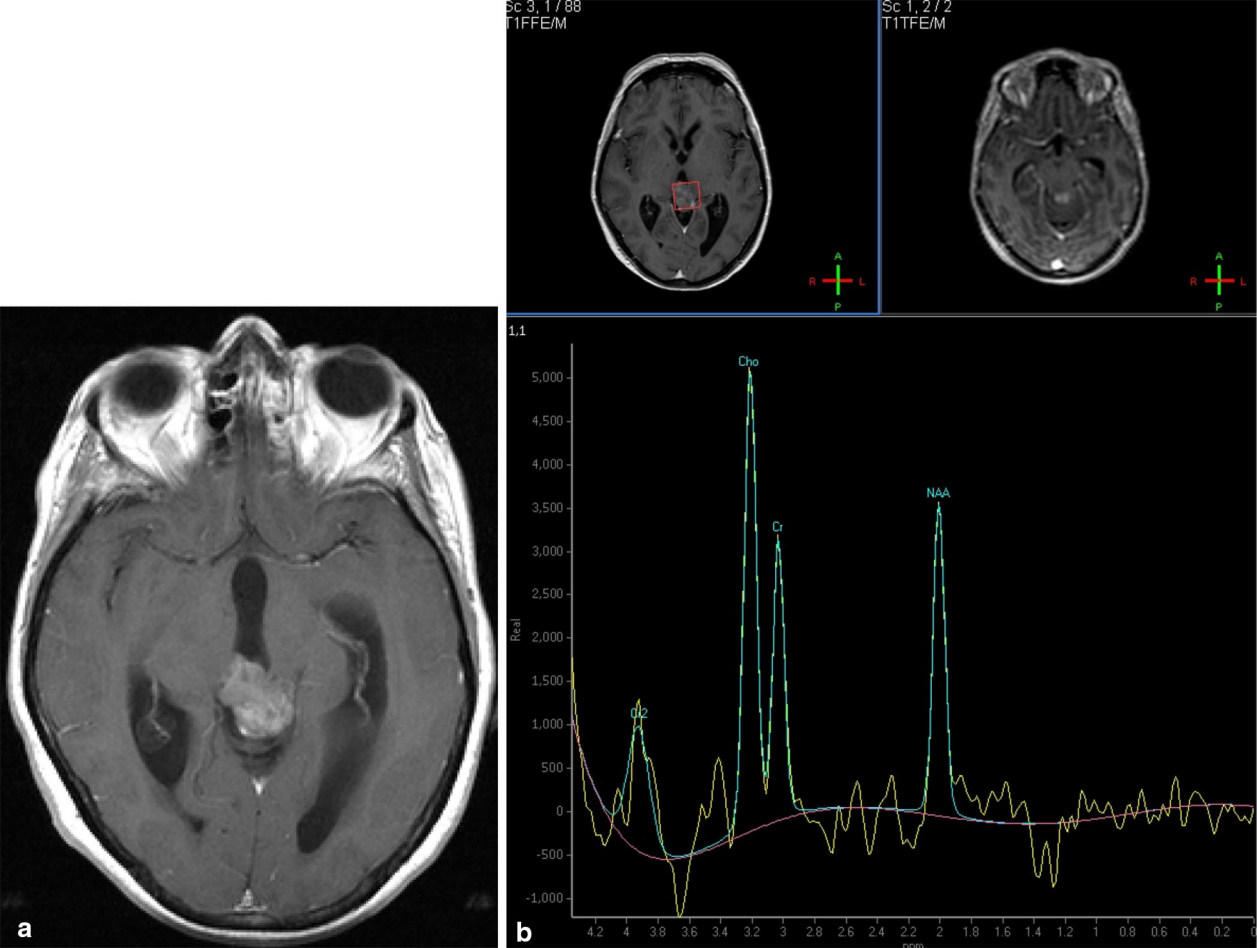
T1-weighted MRI scan after intravenous Gadolinium **a** shows patchy enhancement of mass in pineal region and obstructive hydrocephalus. No evidence of local invasion. **b** Single-voxel MR spectroscopy (TE = 144) shows moderate elevation of Choline, reduction in NAA and inverted lactate doublet

### Histopathology

Pineal parenchymal tumours of intermediate differentiation are graded as lying between Pineocytomas (WHO I) and Pineoblastomas (WHO IV). Pineocytomas are well differentiated with small, uniform, mature cells and virtually lack mitoses. They have numerous, large pineocytomatous rosettes. Pineoblastomas are highly cellular tumours with frequent mitotic figures, irregular nuclei, large nucleus to cytoplasmic ratio; they form pattern-less sheets, with necrotic areas being common, and rosettes are rare (Louis et al. 2007). Pineal parenchymal tumours of intermediate differentiation are still a grey area but generally have moderate cellularity, mild to moderate nuclear atypia and low to moderate mitotic activity. Necrosis and endothelial proliferation are absent in contrast to pineoblastomas (Mena et al. 2000).

**Table 2:** Different published studies used radiotherapy for PPTID

Authors	Total Number of patients in the study	Total number of patients with radiotherapy	Number of patients with Craniospinal radiotherapy	Dose	Stereotactic	Chemotherapy	Outcome
Schild et al. (1993)	4	4	Not clear	45–64.8 Gy	Not clear	Not clear	No specific data
Fauchon et al. (2000)	26 patients were classified as Grade 2 and Grade 3 not PPID	14	12	Neuroaxis Dose average 31 Gy (range 10–38 Gy)	Yes	Yes 6 patients Various regime	5 years survival 74 % and 39 % for Grade 2 and 3 respectively
Lutterbach et al. (2002)	37 specimens were retrospectively analysed to classify them as PPTIDs	Not clear	Not clear	Range 20–75 Gy Median 54 Gy	Yes	Yes Cis/Vin, Cis/Eto, Cap/Ifo, VIP16/Cis, Vip16/D/F, MTX it, LO/CIS/VI	Median overall survival 165 months
Stoiber et al. (2010)	1	1	0	54 Gy/30 f L	No	No	Time to progression 84 months
Tsubasa et al. (2014)	5	5	2	L 54 Gy, CSI 36 Gy WVI 18	No	Yes 4 patients VNCI	Medial overall survival 94.1 months
Ito et al. (2014)	6	6	4	22 Gy/10 f L 54.4 Gy/28 f WB + L+WS 50 Gy/25 f EL+L	Yes	Yes 3 patients ACNU,VDS,VCR,ICE	Median event free survival 39 months

*L* local, *EL* extended local, *WB* whole brain, *WS* whole spine, *WVI* whole ventricular irradiation, *VNCI* vincristine, nimustine, carboplatin, interferon, *CSI* craniospinal irradiation, *ACNU*, 1-(4-amino-2-methyl-5-pyrimidinyl)methyl-3-(2-chloroethyl)-3-nitrosourea hydrochloride, *VDS* vindesine, *VCR* vincristine; *ICE* ifosfamide + *cis*-platinum + etoposide, *Cis* cisplatinum, *Vi* vincristine, *VP16* etoposide, *MTX* methotrexate, *l0* Lomustiene

The presence of necrosis, mitotic rate and immunohistochemical expression of neurofilament protein are used to classify PPTIDs as grade II or III pathologically (Sato and Kubota 2009). Jouvet et al. defined that grade 2 tumour has <6 mitoses and strongly immunopositive for neurofilaments and grade 3 will have, >6 mitoses or <6 mitoses, but without strong immunostaining for neurofilaments (Jouvet et al. 2000).

Tsumanuma et al. described MIB-1 index as significantly higher in PB after analysing 13 cases of Pineal Parenchymal tumours (Tsumanuma et al. 1999). A clinicopathologic study of PPTID by Ito et al. found MIB-1 level correlates with WHO grade (Ito et al. 2014).

Figures 1 (grade II) and 2 (grade III) describe the histopathological characteristics of two of our patients. In these two cases, the tumours are more cellular than in pineocytoma and the cells have more atypical nuclei but not atypical enough to be labelled pineoblastoma. Both cases were Synaptophysin positive, which support a tumour of pineal origin.

Mitotic figures are seen (more easily identified in the grade III case) which is uncharacteristic of a pineocytoma but the low MIB-1 proliferation index would weigh against a pineoblastoma. The Fig. 1 also shows very strong positivity towards neurofilament staining.

### Radiological diagnosis

Radiologically, PPTID is difficult to differentiate from other pineal tumours (Osborn et al. 2012; Fang and Meyers 2013). Peripheral displacement of pineal calcification may help to confirm a tumour of pineal parenchymal origin (Osborn et al. 2012). Both pineoblastoma and PPTID may show local invasion, but pineoblastoma is typically seen in children. PPID is distinguished from pinocytoma by large size and focal invasion of adjacent structures. Hemorrhage and cysts are common (Fig. 3). Therefore, it has been suggested that the Radiologist should suggest PPTID if a locally invasive enhancing pineal parenchymal tumour is seen in an adult patient. However, this finding was only seen in one of our patients, although it is interesting that this tumour also demonstrated diffusion restriction which can be a marker of cellular and more anaplastic tumours (Cha 2006). Magnetic resonance spectroscopy in one of our patients showed changes consistent with tumour, but not specific for PPTID (Fig. 4).

### Treatment

Four patients had an R2-resection due to tumour location and invasion toward the surrounding eloquent areas. One patient had biopsy only. No post operative complication was documented. All the patients were treated with external beam radiation therapy post operatively.

#### Radiotherapy

All patients were treated in supine position and immobilised with Beam Directed Shell (BDS). Planning CT scan was fused with post operative MRI scan to delineate tumour volume. Gross Tumour Volume (GTV) was defined as all visible tumour on post operative MRI scan and planning CT scan. Clinical Target Volume (CTV) was created by adding a 15 mm margin to the GTV. CTV to Planning Treatment Volume (PTV) margin was 5 mm. The prescribed dose was 54 Gy in 30 fractions (1.8 Gy per fraction). The radiation was delivered by a linear accelerator with energy of 6 MV. Patients were assessed for side effects and compliance with treatment on a weekly basis during therapy. Patients underwent 3 monthly follow-up MRI scans in the first year and then 6 monthly thereafter.

## Outcome

### Result

**Fig. 5:**
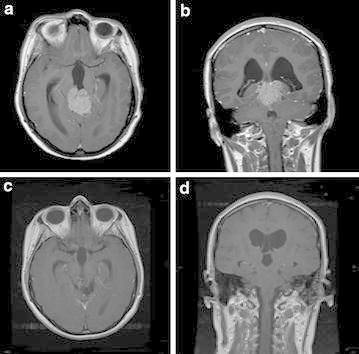
Patient with good PR (partial response). Axial and coronal T1-weighted MRI scans with intravenous Gadolinium, before (**a** and **b**) and after (**c** and **d**) treatment

No patients developed recurrence after 21.4 months of median follow up (range 7–43 months). Four patients achieved PR (Fig. 5) and 1 patient had SD. All patients were alive at the end of the observation period.

### Toxicity

All patients had an uneventful post-operative recovery. No serious intracranial bleeding was recorded in the perioperative period. No patients exhibited evidence of neurocognitive disorders, neither was there any evidence of cerebral white matter abnormalities on follow up MRI scans. So far no endocrine deficiency or visual impairment has been documented.

## Discussion

There is limited evidence available to guide the management of PPTID and currently no general consensus exists concerning treatment. Our study may add important evidence regarding viable treatment outcomes with localised field radiation therapy in PPTID.

### Radiotherapy

PPTID is radiosensitive tumour (Anan et al. 2006). Rickert et al. reported that PPTIDs resembled PB genomically but PC prognostically, which raised an important question of whether PPTIDs should be treated in a similar fashion to PC or PB (Rickert et al. 2001).

The role of radiotherapy has been highlighted in various studies but the extent of radiation therapy in PPTID has yet to be determined. The role of craniospinal and whole-ventricular irradiation for patients with PPTID remains controversial. A single institutional study of six patients with PPTID by Ito et al. showed five cases obtained almost complete response to radiotherapy but only 2 patients had localised radiotherapy (Ito et al. 2014). Lutterbach et al. pointed out that survival outcome is strongly related with CSF dissemination in adult pineal parenchymal tumours and spinal control was more successful in patients with pineal parenchymal tumours of intermediate differentiation (Lutterbach et al. 2002). Interestingly a clinicopathological study of 76 patients with pineal tumour revealed extent of radiotherapy had no clear influence on survival (Fauchon et al. 2000). Stoiber et al. reported local radiotherapy seems to be effective in patients with PC and some PPTIDs after analysing patients retrospectively (Stoiber et al. 2010).

Ito et al. has raised questions whether radiotherapy could be omitted in grade 2 PPTID? (Ito et al. 2014). We felt postoperative RT is necessary for all cases as complete resection is extremely difficult due to the invasive nature of the disease.

The association between radiation dose and survival outcome is also a matter of debate. A study by Schild et al. (1996) of 30 patients showed an link between the radiation dose and survival time in patients with pineal parenchymal tumours. In that study patients who received doses >50 Gy had a significantly improved 3-year survival rate compared to those who received lower doses (94 vs 56 %, respectively) .In our study all patients were treated with 54 Gy to primary site with good outcome which is consistent with Schild’s report. Table 2 summarises various published studies which used radiotherapy in PPTID.

Interestingly all patients in our cohort were treated with localised radiotherapy with no relapses to date. Craniospinal radiation would be a logical approach for patients with spinal dissemination. Our experience raised an important question should we exclude spinal radiation for patient who do not have spinal involvement radiologically?

### Chemotherapy

The role of chemotherapy in PPTID patients remains controversial. A retrospective review of PPTID patients by Tsubasa et al. showed four out of five patients received six courses of combination chemotherapy with vincristine (0.6 mg/m^2^), nimustine (60 mg/m^2^), carboplatin (110 mg/m^2^) and interferon β (3 × 10^6^ IU) (Tsubasa et al. 2014). One patient, who did not receive chemotherapy, developed spinal seeding after treatment. Another study utilised combination chemotherapy with cisplatin and vinblastine as systemic treatment of pineal parenchymal cell tumours (Kurisaka et al. 1998). Li et al. demonstrated the presence of a mutation of epidermal growth factor receptor (in-frame deletion of exons 2–7) in PPTID tumours (Li et al. 2010). This study indicated the probable benefit of targeted agents in PPTID. Interestingly our study would call into question the routine use of chemotherapy in PPTID patients with localised disease given the excellent outcome to date.

Due to long survival outcome of PPTID patients, late adverse effects are an important consideration. Wide field irradiation with the addition of systemic therapy definitely increases the long term side effects of treatment. The study by Tsubasa showed two patients who received craniospinal irradiation exhibited severe cognitive impairment 4–6 years after radiation therapy (Tsubasa et al. 2014). Some data suggested injury to neural progenitor cells plays an important role in treatment-related neurocognitive toxicity (Limoli et al. 2004).

Chemotherapy also enhances neurocognitive toxicity due to the different sensitivity of normal neural stem cells (Gong et al. 2011). Targeted agents, including epidermal growth factor receptor tyrosine kinase inhibitors and proteasome inhibitors, were also found to be potentially more neurotoxic compared to conventional chemotherapeutic agents (Bota et al. 2013). Radiation contributes to increased chemotherapeutic neurocognitive toxicity due to blood brain barrier damage which essentially increases the penetration of chemotherapy (Brown et al. 2007).

There are some limitations to our study. Firstly, It is a retrospective study with a small number of patients and consequently care must be taken in generalising our results to a larger population. Secondly, follow-up is short at under 2 years, particularly if long-term toxicity is to be reliably estimated. In addition we could be criticised for failing to perform routine pre-operative CSF (cerebro spinal fluid) analysis to exclude the presence of CSF seeding particularly as we are advocating a localised radiotherapy approach to treatment.

## Conclusion

Our experience indicates that localised radiation therapy could be an effective treatment strategy for PPTID, considering the long natural course of the disease and the late adverse effects of intensive treatment. We did not use spinal radiation and systemic therapy in an attempt to minimise treatment related toxicities and none of our patients has experienced disease recurrence to date. Taking into account the rarity of this disease, prospective multi-institutional studies could be extremely difficult. One realistic option would be to consider a registry based study.
